# Transcriptome Analysis of PPARγ Target Genes Reveals the Involvement of Lysyl Oxidase in Human Placental Cytotrophoblast Invasion

**DOI:** 10.1371/journal.pone.0079413

**Published:** 2013-11-12

**Authors:** Nadine Segond, Séverine A. Degrelle, Sarah Berndt, Elodie Clouqueur, Christine Rouault, Bruno Saubamea, Philippe Dessen, Keith S. K. Fong, Katalin Csiszar, Josette Badet, Danièle Evain-Brion, Thierry Fournier

**Affiliations:** 1 INSERM, UMR-S767, Paris, France; 2 Université Paris Descartes, Sorbonne Paris Cité, Paris, France; 3 PremUP Foundation, Paris, France; 4 INSERM, UMR 872, Equipe 7, Paris, France; 5 Université Pierre et Marie Curie, Paris, France; 6 INSERM, U705, Paris, France; 7 CNRS, UMR 8206, Paris, France; 8 INSERM, UMR 985, Institut Gustave Roussy, Villejuif, France; 9 John A. Burns School of Medicine, University of Hawaii, Honolulu, Hawaii, United States of America; Brigham and Women's Hospital, United States of America

## Abstract

Human placental development is characterized by invasion of extravillous cytotrophoblasts (EVCTs) into the uterine wall during the first trimester of pregnancy. Peroxisome proliferator-activated receptor γ (PPARγ) plays a major role in placental development, and activation of PPARγ by its agonists results in inhibition of EVCT invasion *in vitro*. To identify PPARγ target genes, microarray analysis was performed using GeneChip technology on EVCT primary cultures obtained from first-trimester human placentas. Gene expression was compared in EVCTs treated with the PPARγ agonist rosiglitazone versus control. A total of 139 differentially regulated genes were identified, and changes in the expression of the following 8 genes were confirmed by reverse transcription-quantitative polymerase chain reaction: a disintegrin and metalloproteinase *domain12 (ADAM12)*, connexin 43 (*CX43)*, deleted in liver cancer 1 (*DLC1)*, dipeptidyl peptidase 4 (*DPP4)*, heme oxygenase 1 (*HMOX-1)*, lysyl oxidase (*LOX)*, plasminogen activator inhibitor 1 (*PAI-1)* and *PPAR*γ. Among the upregulated genes, lysyl oxidase (*LOX*) was further analyzed. In the LOX family, only LOX, LOXL1 and LOXL2 mRNA expression was significantly upregulated in rosiglitazone-treated EVCTs. RNA and protein expression of the subfamily members LOX, LOXL1 and LOXL2 were analyzed by absolute RT-qPCR and western blotting, and localized by immunohistochemistry and immunofluorescence-confocal microscopy. LOX protein was immunodetected in the EVCT cytoplasm, while LOXL1 was found in the nucleus and nucleolus. No signal was detected for LOXL2 protein. Specific inhibition of LOX activity by β-aminopropionitrile in cell invasion assays led to an increase in EVCT invasiveness. These results suggest that *LOX*, *LOXL1* and *LOXL2* are downstream PPARγ targets and that LOX activity is a negative regulator of trophoblastic cell invasion.

## Introduction

**Figure 1:**
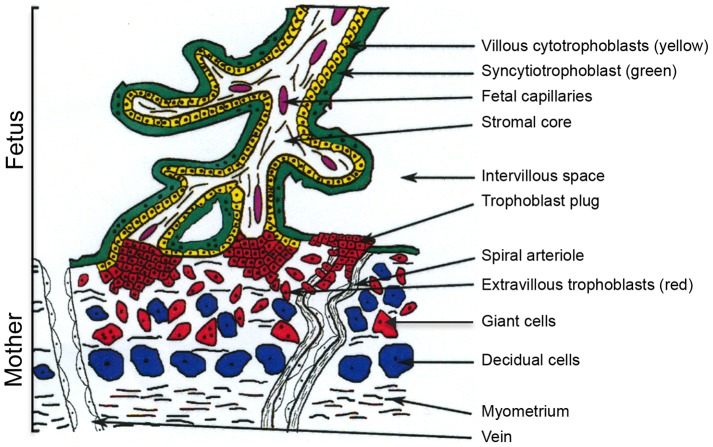
Representation of a chorionic villus at the implantation site. Villous cytotrophoblasts (yellow) fuse to form the syncytiotrophoblast (green). The extravillous trophoblasts (red) proliferate to form multilayered columns of cells and then invade the decidua up to the upper third of the myometrium and the uterine arterioles. At the deciduo-muscular junction, EVCTs undergo final differentiation into multinucleated giant cells. Adapted from figure 1 of Tarrade *et al.* [12].

Human placental development relies on trophoblast differentiation along two pathways. Villous cytotrophoblasts (VCTs) fuse to form a syncytiotrophoblast (ST) involved in placental exchanges and endocrine function, while extravillous trophoblasts (EVCTs) anchor the chorionic villi in the maternal uterus. A subpopulation of EVCTs ceases to divide and invades the uterine wall as far as the innermost third of the myometrium and the maternal spiral arteries [1], [2] (Figure 1). This invasion coincides with remodeling of arterial walls, resulting in low-resistance blood vessels providing optimal maternal-fetal exchanges. Limited maternal perfusion of the intervillous space, together with histiotrophic nutrition from uterine glands, protects the fetus from high oxygen tension during these early stages of differentiation [3], [4]. Trophoblast plugging of the maternal spiral arteries between 6 to 8 weeks of gestation is gradually eliminated between 8 to 12 weeks of gestation, leading to increases in intervillous oxygen tension and placental expression of anti-oxidant enzymes [5], [6]. This physiological trophoblastic cell invasion process is tightly controlled during the first trimester and is required for placental development and normal pregnancy outcome. Indeed, impaired trophoblast invasion has been implicated in gestational pathologies such as fetal growth restriction and preeclampsia [7], [8], [9].

To study early human placental development and the regulation of the trophoblastic cell invasion process, we have developed an *in vitro* invasion model using non proliferative and highly invasive EVCT primary cells isolated from first-trimester human chorionic villi cultured on Matrigel™ [10], [11], [12]. These purified primary EVCTs express *in vitro* the specific markers of human invasive EVCTs described *in situ*, namely cytokeratin 7 [13], [14], [15], human leukocyte antigen-G [16], human placental lactogen [17], c-erbB2 [18] and the alpha 5 subunit of the fibronectin receptor 5ß1 [19]. The limiting step of this unique human primary culture model is the low number of EVCTs that can be isolated from early first-trimester placental tissue (8–9 weeks of amenorrhea, WA).

Using this model, we have previously shown that peroxisome proliferator-activated receptor γ(PPARγ) activation by the synthetic and specific agonist rosiglitazone [20] inhibits EVCT invasiveness in a concentration-dependent manner, reaching statistically significant 50% inhibition at a concentration of 1 µM [12]. PPARγ, a member of the ligand-activated nuclear receptor superfamily, controls the expression of many genes involved in metabolism, cell differentiation and tumorigenesis. DNA binding of PPARγ to its response element PPRE (composed of a direct repeat of the core hexanucleotide motif AGGTCA with one intervening base named DR1) requires heterodimerization with another nuclear receptor, the retinoid X receptor (RXR) (for review see [21]). In mice, PPARγ gene inactivation results in abnormal placental development, with defects in trophoblast differentiation and vascular processes leading to embryonic lethality at E10 [22], [23]. However, PPARγ expression in trophoblasts is sufficient to rescue *PPARγ^−/−^* embryonic lethality [24], [25]. These studies demonstrated that trophoblastic expression of PPARγ is essential for implantation and for the formation of a functional placenta in mice. In the human placenta, PPARγ is exclusively located in the nuclei of villous trophoblasts throughout pregnancy and, from first trimester placentas, in extravillous trophoblasts. PPARγ is thus a trophoblast-specific marker that can be immunodetected in cytokeratin 7-positive VCT, ST and EVCT throughout differentiation [12], [26], [27], [28].

Here we used our *in vitro* model of invasive primary EVCTs to identify genes involved in PPARγ-mediated trophoblast invasion, based on a transcriptomic approach. Expression of numerous genes was modulated by rosiglitazone treatment of EVCTs isolated from 8–9 WA placentas. To confirm the transcriptome results, we used RT-qPCR to analyze eight PPARγ-target genes that were the most strongly modified and/or were potentially involved in EVCT invasion, such as dipeptidyl peptidase 4 (*DPP4*), heme oxygenase 1 (*HO-1*, *HMOX1*), connexin 43 (*CX43*, *GJA1*), plasminogen activator inhibitor 1 (*PAI-1*, *SERPINE1*) and lysyl oxidase (*LOX*, EC 1.4.3.13).

LOX is a copper-dependent monoamine oxidase known to catalyze the formation of covalent cross-links of lysine residues within components of the extracellular matrix (ECM), including fibrillar collagen and elastin [29]. Molecular oxygen is necessary to complete the catalytic cycle of LOX, with the release of hydrogen peroxide and ammonia [29]. LOX expression and activity are both dependent on oxygen levels [30]. Like LOX, the four members of the LOX-like family (LOXL1, LOXL2, LOXL3 and LOXL4) display catalytic activity and have both ECM and cellular functions [31]. They all contain a signal peptide and a putative PPARγ response element (Genomatix Software GmbH, http://www.genomatix.de, accessed 2013). These five isoforms can be divided into two subgroups according to their sequence, structure and processing. LOXL2, LOXL3 and LOXL4 contain four scavenger receptor cysteine-rich (SRCR) domains, but little is known of their processing. LOX and LOXL1 are largely homologous, do not contain a SRCR domain, and are secreted as proproteins; this is the subfamily we studied in invasive EVCTs – LOXL2 was also considered.

Among the trophoblastic PPARγ target genes revealed by our transcriptomic approach, we focused on the expression and location of the LOX, LOXL1 and LOXL2 isoforms in early first-trimester human placental tissues *in situ* and in primary invasive EVCTs *in vitro*. The role of these isoforms in EVCT invasion was addressed in experiments using their specific catalytic inhibitor β-aminopropionitrile (BAPN) [31] and our primary culture model.

## Results

### Gene expression profiling of rosiglitazone-treated trophoblasts

**Figure 2:**
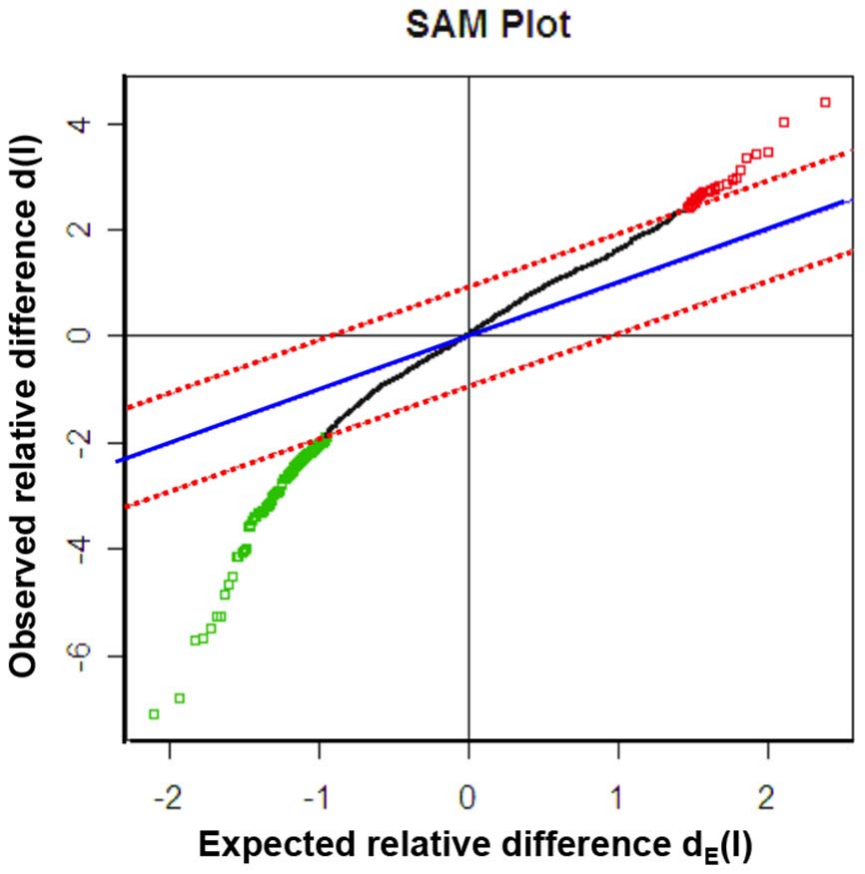
Microarray and transcriptome analyses of rosiglitazone-treated EVCTs purified from first-trimester placentas. SAM scatter plot of the observed d(l) versus the expected d_E_(l) relative difference between rosiglitazone-treated cells and their paired controls (n = 5), using a two-fold threshold. EVCTs were cultured for 24 h and then treated with rosiglitazone for 24 h. Gene profiles were compared with those of paired untreated control EVCTs from the same placenta. The solid blue line represents no difference between d(l) and d_E_(l), and the broken red lines show the delta limit of 0.93 from the solid blue line. A total of 175 probe sets outside the broken lines were significantly induced (red dots, n = 149) or suppressed (green dots, n = 26) in rosiglitazone-treated cells. The median estimated FDR was <5%.

**Figure 3:**
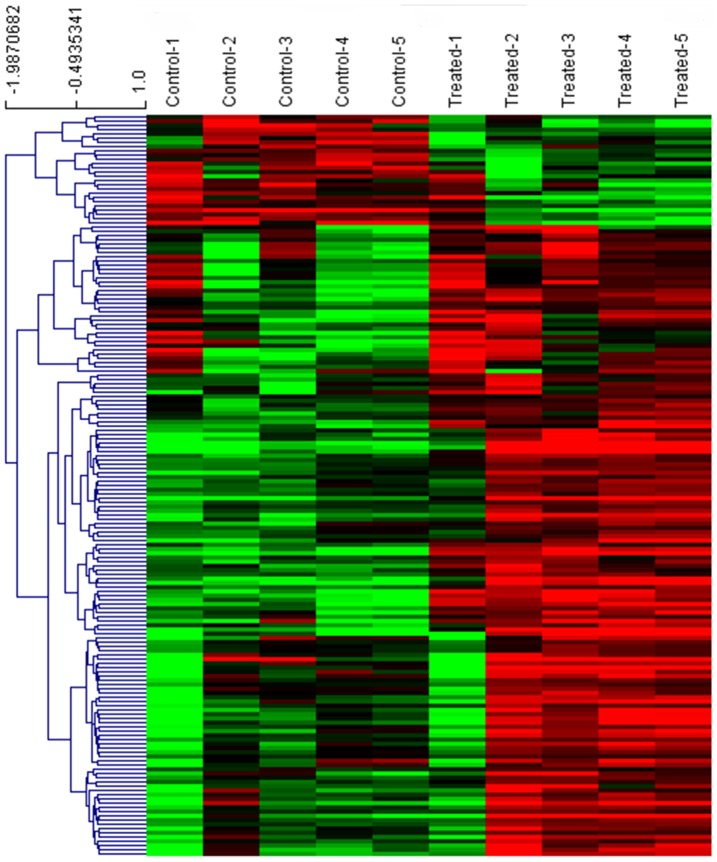
Heatmap of the 175 probe sets (117 unique genes) selected with the SAM procedure. Each column represents an individual sample (5 controls and 5 samples treated with 1 µM rosiglitazone). Each row represents one probe. The gene expression level is depicted by the intensity of the green (low intensity) and red (high intensity) boxes. Probe and gene names are included in Figure S1.

**Figure 4:**
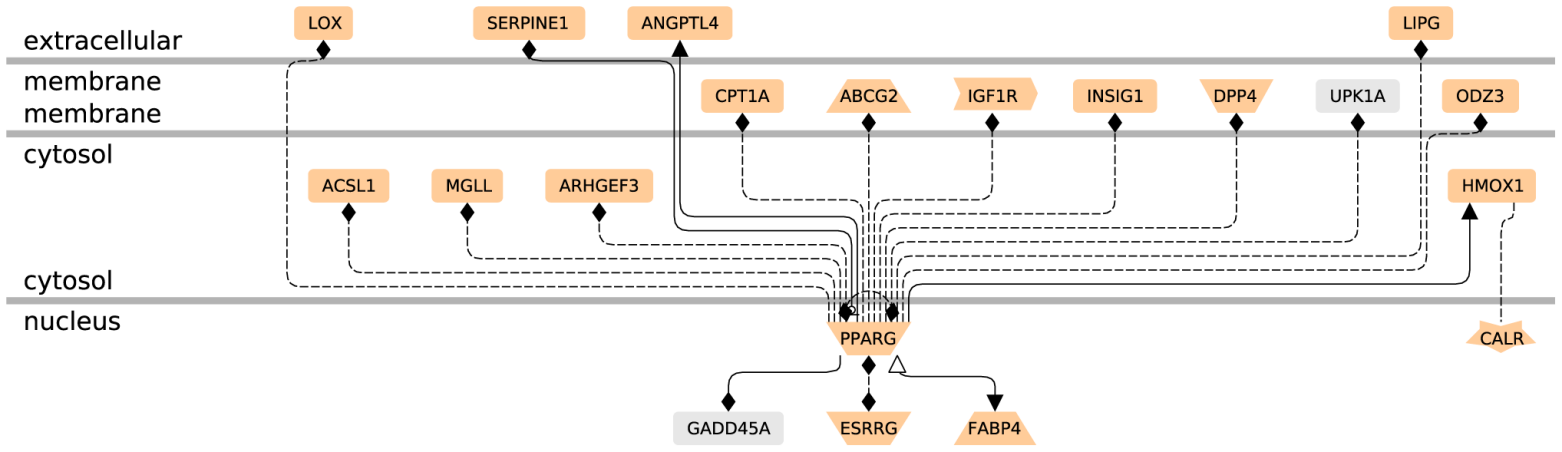
Networks of the 20 differentially expressed genes containing putative PPARγ response elements (PPREs) in their promoter. The entire 117 unique gene list was uploaded into the Genomatix Pathway System (GePS 2.4.0). Only the 20 genes linked to PPARγ by a PPRE are represented. The function of each gene is that proposed by GePS software for receptors and co-factors, etc. (for further details see the Genomatix web site; http://www.genomatix.de). The pale salmon color represents genes expressed in human placenta (Unigene): 2 genes (*UPK1A* and *GADD45A*) are not mentioned as being expressed in human placenta on this web site, but GADD45A expression has recently been described in this tissue [100].

Gene profiling of EVCTs isolated from first-trimester human placenta, with comparisons of rosiglitazone-treated and control cells from the same placenta, was performed with an Affymetrix GeneChip analyzing 14 500 genes with 22 000 probe sets. The microarray data are available in the gene expression omnibus database (http://www.ncbi.nlm.nih.gov/geo, 2012 GSE28426). Mean results are shown for rosiglitazone-treated cells from five different placentas *vs* their paired controls (SAM scatter plot in Figure 2, heat map in Figure 3 and Figure S1). Five independent EVCT cultures yielded similar results in four cases and a slightly different pattern for culture 1. A total of 139 genes (175 probe sets, 117 unique genes) were identified as having significantly different expression (p<0.05) in treated EVCTs. Overall, 114 genes (149 probe sets) were over-expressed (red) and 25 genes (26 probe sets) were under-expressed (green) in treated EVCTs. The complete gene list is presented in Table S1. Of the 20 genes possessing a putative PPARγ response element (Genomatix Software GmbH, http://www.genomatix.de, accessed 2013), 17 are upregulated and 3 downregulated (Figure 4). All of them are reported to be expressed in the placenta, except for *UPKIA*, initially described as being involved in urothelial cell differentiation [32].

### Functional network analyses

**Figure 5:**
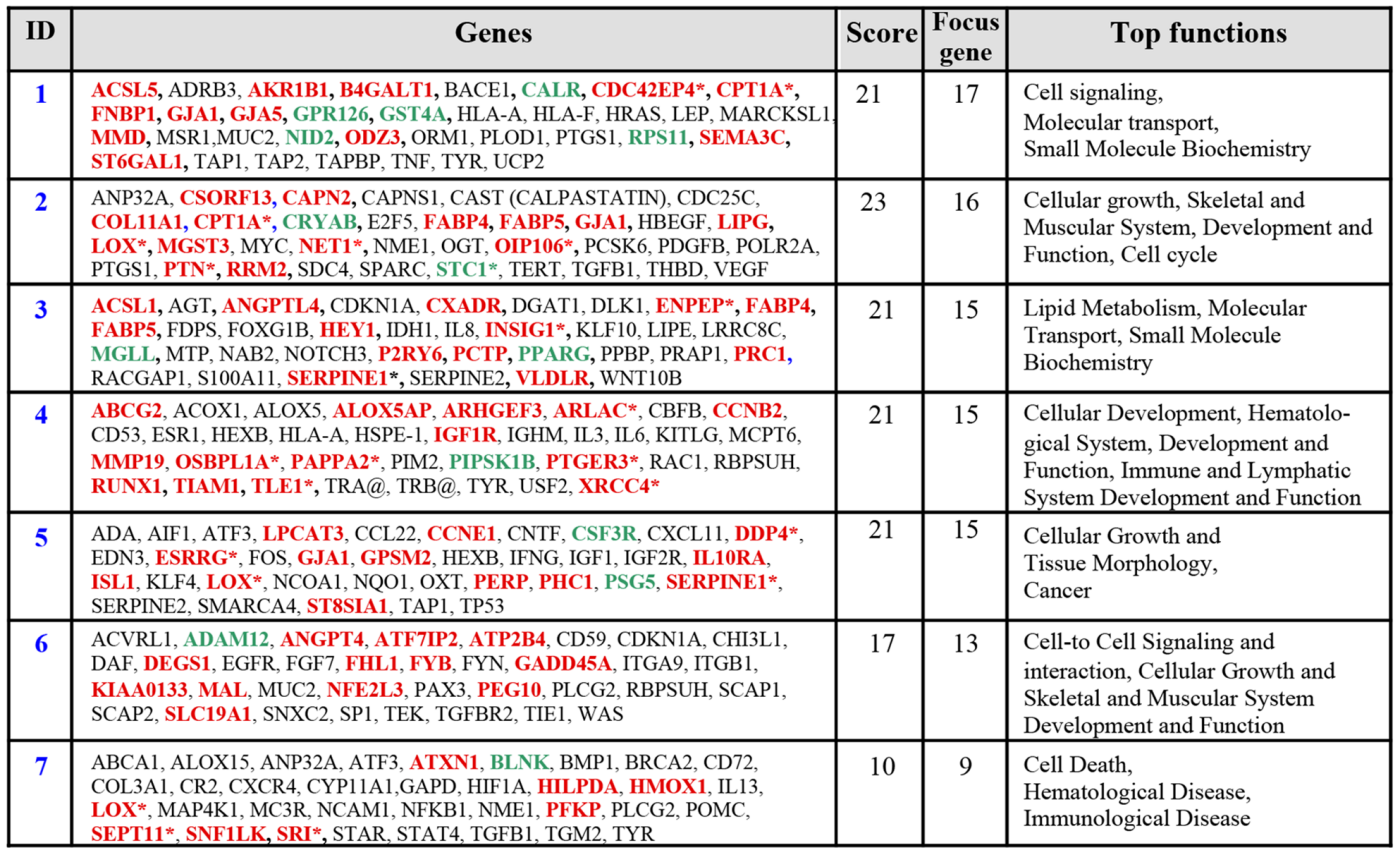
Microarray and transcriptome analyses of rosiglitazone-treated EVCTs compared to paired controls: top 7 networks. The 175 probe sets were loaded into Ingenuity Pathway Analysis software (IPA) and converted into gene networks. Genes shown in bold type are present in the input data list as upregulated (red) or downregulated (green). Genes involved in the network that are not included in the transcriptome results are shown in black. * genes with several probes present in the input data list.

Expression profiles were analyzed with Ingenuity Pathway Analysis software. The top seven functional networks showing altered gene expression in rosiglitazone-treated EVCTs are shown in Figure 5. Functional areas included cell signaling, molecular transport and small-molecule biochemistry, cell growth, lipid metabolism, cell cycling, tissue morphology, skeletal and muscular system development and function, cancer, and cell death. The *LOX* gene was found to be part of networks 2, 5 and 7 (Figure 5).

**Figure 6:**
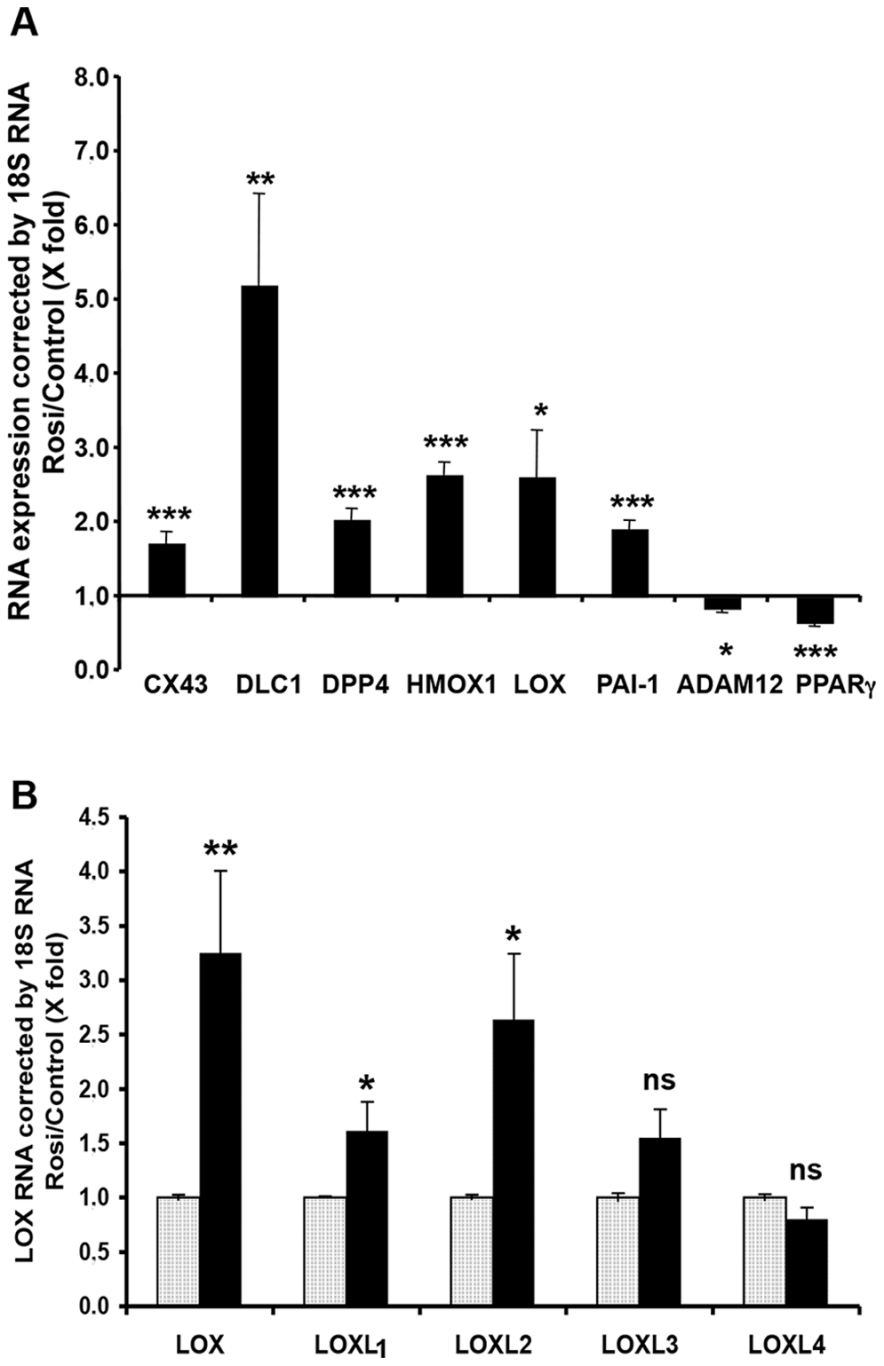
Relative RT-qPCR analysis of the effect of rosiglitazone on EVCT gene expression. EVCTs were purified and treated with rosiglitazone in an identical manner as for microarray analyses. RNA expression estimated with relative RT-qPCR is shown **A**) for selected genes identified as differentially regulated by GeneChip analyses; and **B**) for the five known LOX isoforms. Five treated cultures (black bar) were normalized to control cultures (gray bar) from the same placenta. Results are expressed as the mean ± SEM of six (A) and five experiments (B) performed in duplicate. * p<0.05, ** p<0.01, *** p<0.001 versus untreated cultures; ns: not significant.

The transcriptome data were confirmed for selected genes, namely those found here to be strongly regulated or known to be involved in placental development. RT-qPCR was applied to treated and paired control EVCT cultures distinct from those used for the microarray experiments (Figure S2). RNA levels of connexin 43 (*CX43*, *GJA1*), deleted in liver cancer 1 (*DLC1*), dipeptidyl peptidase 4 (*DPP4*), heme oxygenase 1 (*HO-1*, *HMOX1*), lysyl oxidase (*LOX*) and plasminogen activator inhibitor 1 (*PAI-1*, *SERPINE1*) following 24 h of exposure to rosiglitazone (1 µM) were between 1.7-fold (*CX43*) and 5.2-fold (*DLC1*) higher than in paired untreated cultures, while *ADAM12* and *PPARγ* expression decreased by 0.2- and 0.4-fold, respectively. These differences were statistically significant for all the genes analyzed (*LOX*, *ADAM12*, p<0.05; *DLC1* p≤0.01; *CX43*, *DPP4*, *HO-1*, *PAI-1*, *PPARγ*, p<0.001) and confirmed the GeneChip results (Figure 6A).

### LOX expression in first-trimester EVCTs

Among the genes found to be upregulated in rosiglitazone-treated EVCTs, we further examined the role of LOXs. Both GeneChip and RT-qPCR experiments showed *LOX* to be upregulated in rosiglitazone-treated EVCTs, by 3.3-fold (p<0.05) and 2.6-fold (p<0.05), respectively (Table S1 and Figure 6).

Expression analysis of the *LOX* genes by RT-qPCR showed that rosiglitazone-induced PPARγ activation resulted in a significant increase in the following three of the five LOX isoforms: *LOX* (3.2-fold versus controls, p≤0.01), *LOXL1* (1.6-fold, p<0.05) and *LOXL2* (2.6-fold, p<0.05). No significant change in LOXL3 or LOXL4 RNA levels was observed (Figure 6B).

**Figure 7:**
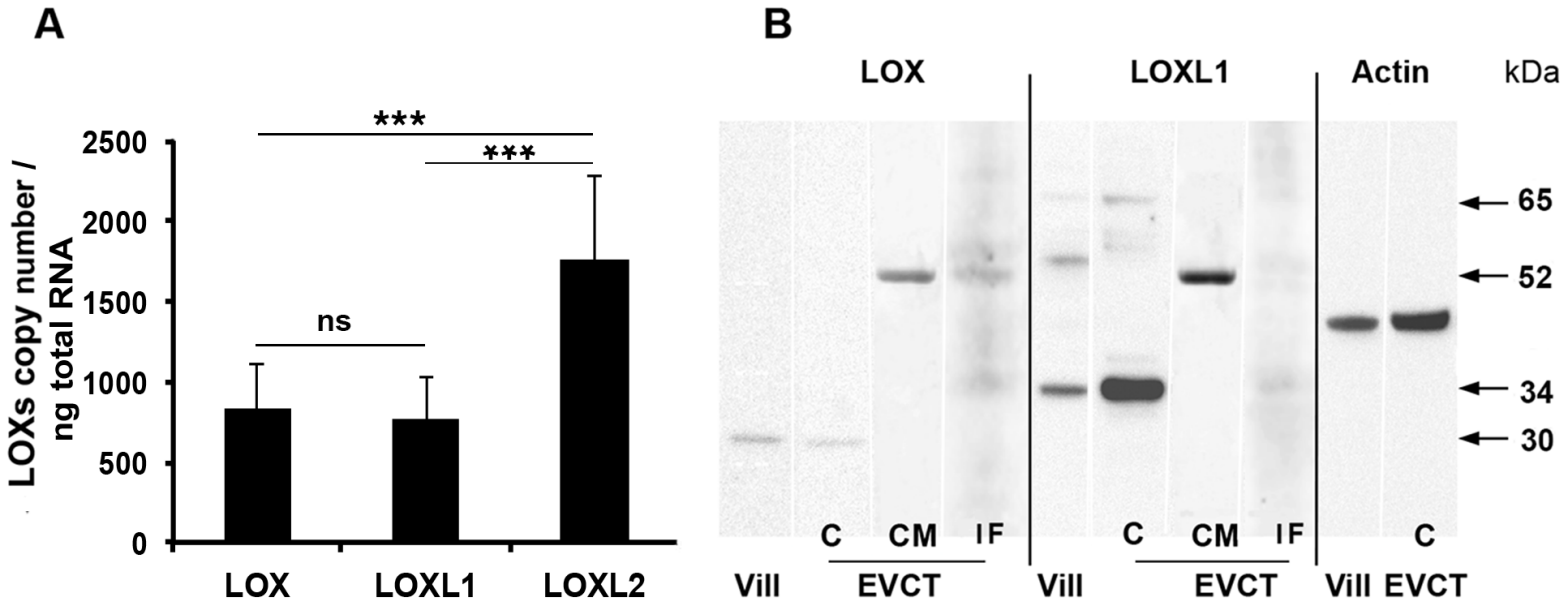
Expression analyses of LOXs in early placental villi and EVCTs. **A**) LOX, LOXL1 and LOXL2 RNA copy numbers per ng of total RNA in 48 h-cultured EVCTs were determined by absolute RT-qPCR. Each bar represents the mean ± SEM of five independent experiments performed in duplicate. *: p<0.05 for differences between isoforms; ns: not significant. **B**) Western blot analyses of LOX and LOXL1 proteins. Protein (20 µg) extracted with the T-PER reagent (Pierce) from 8- to 9-WA placental villous tissue (Vill), or with the M-PER reagent from 72 h-cultured primary EVCTs purified from placental tissue at the same term (EVCT, C), as well as 40 µg of protein from cell conditioned medium (CM) and the insoluble M-PER cell extract fraction (IF) taken up in Laemmli buffer were separated on 4–12% polyacrylamide Bis-Tris gels. Detection was achieved by using LOX and LOXL1 isoform-specific antibodies; ß -actin was used as a control.

We then focused on transcript and protein expression of the *LOX*, *LOXL1* and *LOXL2* genes in placental tissues and control EVCTs. The copy number of each isoform was determined by absolute qPCR in primary EVCT cultures. LOX and LOXL1 RNAs were present in equal quantities (about 800 copies/ng total RNA) (Figure 7A). In first-trimester placental villi and EVCTs, western blot revealed a band at about 30 kDa that might correspond to mature LOX, together with three bands at 34, 52 and 65 kDa that might correspond to mature, pro- and prepro-LOXL1, respectively (Figure 7B). LOX protein was weakly detected in villi and cultured EVCTs. LOXL1 was more abundant (2.3±0.5 fold increase, n = 3, p<0.01) in EVCTs than in villi, suggesting preferential expression of LOXL1 in EVCTs (Figure 7B). Pro-LOX and -LOXL1 were detected in conditioned medium (CM) and in the insoluble fraction of cell extracts (IF). The mature protein was detected in IF at 34 kDa. LOXL2 RNA was expressed in EVCTs (about 1700 copies/ng total RNA) but no significant signal was detected in cells or protein extracts with any of the antibodies tested. An immunofluorescence signal was obtained in term trophoblatic cells, used as a positive control (data not shown).

### Immunolocalization of LOX isoforms in placental tissues and cultured cells

**Figure 8:**
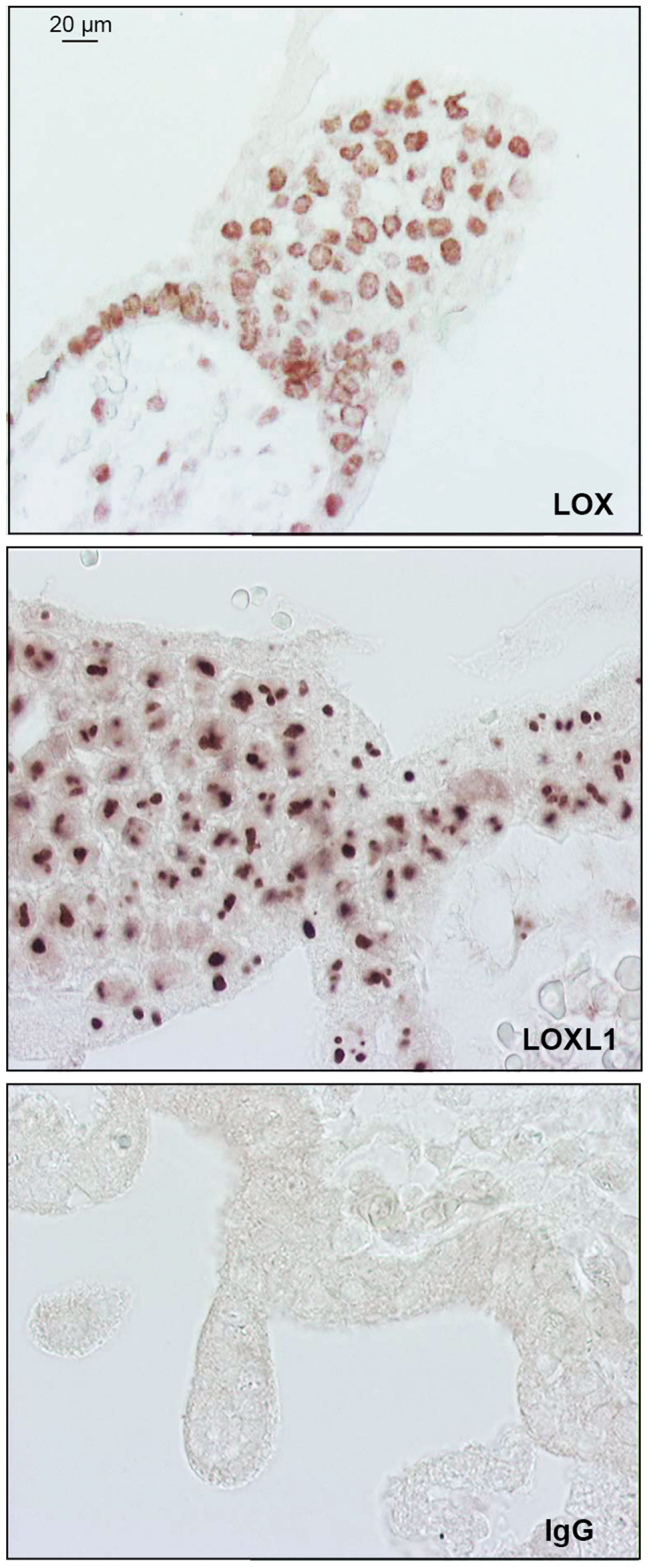
Immunohistological localization of LOX and LOXL1. Experiments were carried out on PFA-fixed sections of 8-WA placental villi, using specific antibodies; non-specific rabbit IgG was used as a control. Immunostaining was performed with an universal streptavidin-peroxidase kit (Dako).

**Figure 9:**
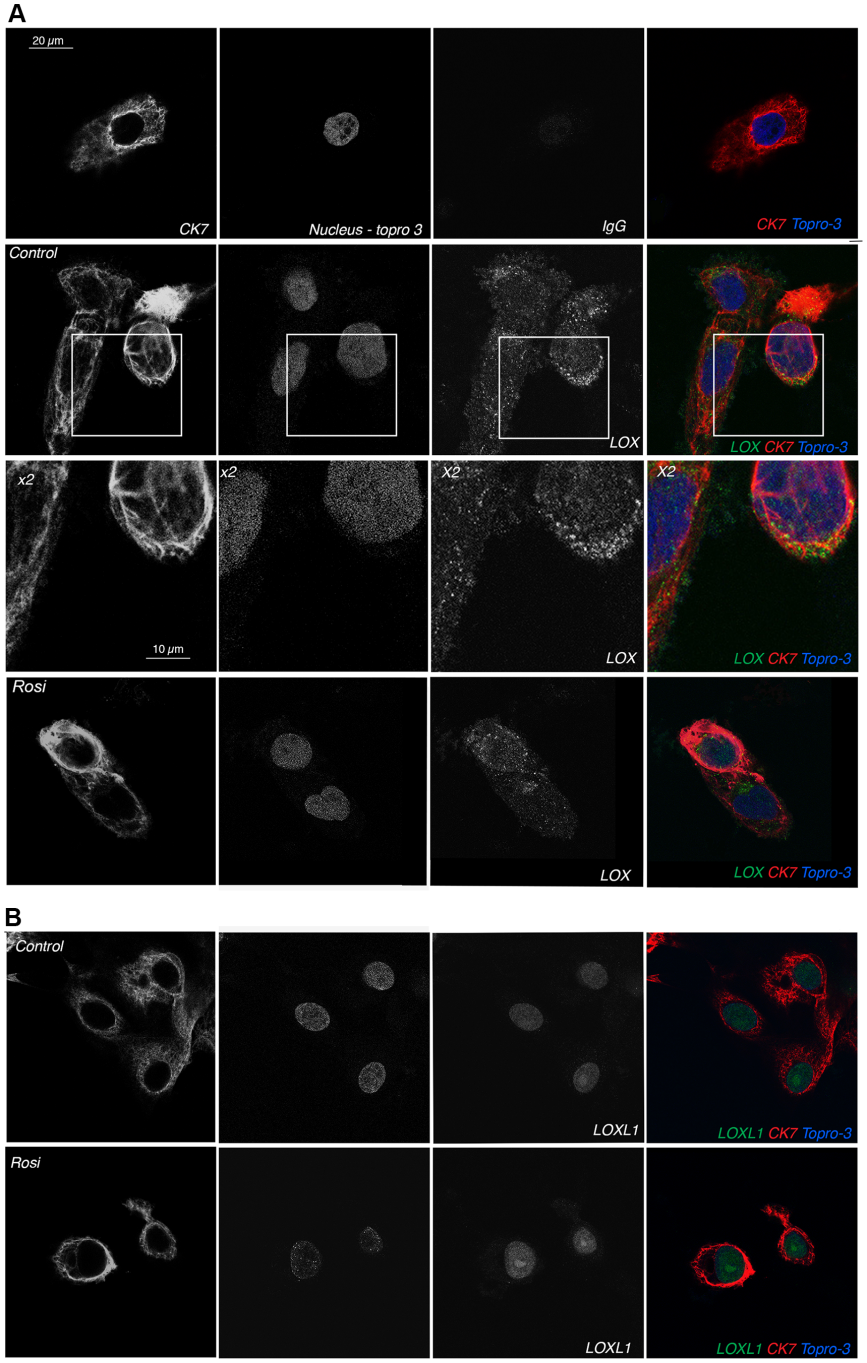
Immunolocalization by confocal microscopy of LOX (Fig 9A) and LOXL1 (Fig 9B) proteins in rosiglitazone-treated and control EVCT primary cultures. EVCTs were cultured for 72™. Anti-LOX and -LOXL1 rabbit antibodies (2.5 µg/mL) were used for immunostaining. Individual staining for CK7 (first column), topro-3 (second column) and LOXs (third column) is shown in grayscale and is merged in the last column; 9A third line: 2× magnification of LOX staining in control cells. LOX isoforms (FITC labeling in green) were detected as follows: LOX in the cytoplasm and around the cells, and LOXL1 mainly in the nucleus and nucleolus. CK7 (CY3 labeling) was used to identify the trophoblast cytoskeleton (in red) and Topro-3 to label the nuclei (in blue). Non-specific rabbit IgG was used as a negative control. The 1 µM rosiglitazone-treated cells (Rosi) were stained in the same conditions. Only the LOXL1 signal was enhanced in rosiglitazone-treated cells.

Immunohistochemical analyses of tissue sections from 8- to 9-WA villous columns showed a cytoplasmic and perinuclear pattern of LOX staining, while LOXL1 was mainly located within nuclei and nucleoli-like structures (Figure 8). Immunofluorescence data for cultured EVCTs were consistent with those obtained by immunohistochemistry. For LOX, immunocytochemistry showed stained spots suggesting the presence of granules throughout the cytoplasm (Figure 9A), whereas LOXL1 was mainly localized in the nucleus, with stronger labeling in the nucleolus (Figure 9B). We also observed pericellular LOX labeling. In rosiglitazone-treated cells we observed an increase in LOXL1 immunolabeling (Figure 9B), as well as cytoskeleton modifications, as shown by CK7 immunostaining (Figures 9A and 9B).

### LOXs affect EVCT invasion

**Figure 10:**
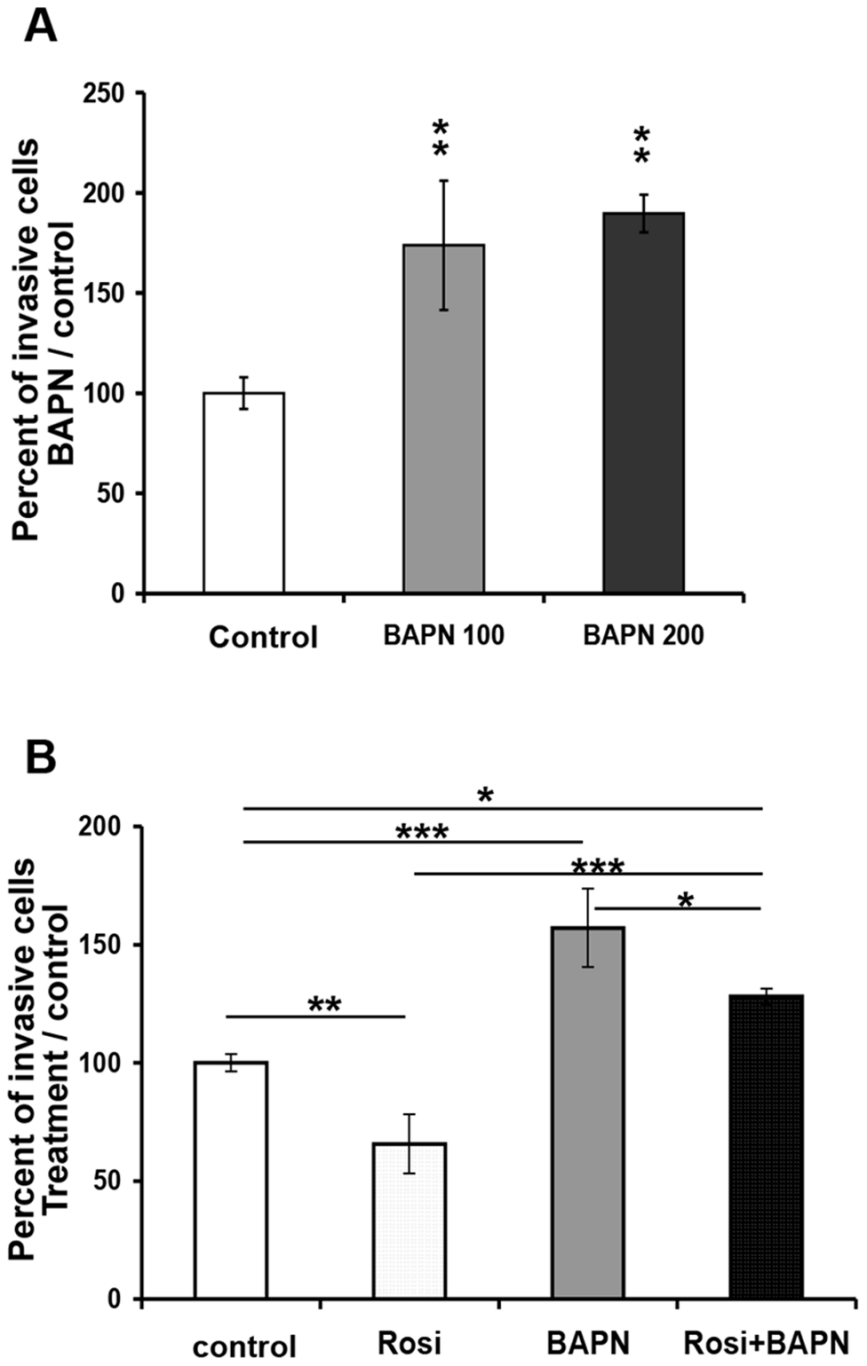
Effect of LOX inhibition on EVCT invasion. EVCTs were isolated from first-trimester placentas, cultured as described in Materials and Methods, and treated 24 h later with **A**) 100 µM or 200 µM BAPN for 48 h or **B**) 1 µM rosiglitazone or 100 µM BAPN, alone or combined, for 48 h. Cells were fixed and stained with DAPI (nuclei) and CK7 (cytotrophoblasts). CK7^+^ migrating cells, nuclei and pseudopodes were counted. The number of invasive EVCTs is expressed as a percentage of the total cell number. Each bar represents the mean ± SEM of four experiments performed in duplicate. Significant differences between treatments are marked as * p<0.05, ** p≤0.01, or *** p<0.001.

We then examined the effect of LOX inhibition on EVCT invasion. LOX activity was inhibited with β-aminopropionitrile (BAPN), a specific and irreversible inhibitor [33]. BAPN-treated EVCTs were nearly twice as invasive as control cells (100 µM, 174±32%; 200 µM, 190 ± 9%; p<0.01; Figure 10A). No significant difference was observed between the effects of 100 and 200 µM BAPN. EVCT invasion was inhibited by 35% (p<0.01) following rosiglitazone treatment, and this effect was overcome by adding BAPN (Figure 10B). The ratios of BAPN-treated/control cells and rosiglitazone+BAPN/rosiglitazone-treated cells were similar, suggesting that the effect of BAPN remained at the same order of magnitude (1.6-fold increase in cell invasion) in the presence and absence of rosiglitazone.

## Discussion

Cell invasion is a fundamental process during embryonic development [34], and also plays an essential role in tumorigenesis [35]. In humans, invasion of cytotrophoblasts into the maternal uterus is a temporally and spatially controlled process critical for successful placental and fetal development [3], [36], [37]. Defective trophoblast invasion has been implicated in complications of pregnancy such as intrauterine growth restriction and preeclampsia [7], [9]. We have previously shown that activation of the nuclear receptor PPARγ by its specific synthetic agonist rosiglitazone and its natural ligands (15-deoxy-Δ-(12,14)prostaglandinJ_2_ (15D-PGJ_2_), 15-hydroxy-eicosatetraenoic acid (15-HETE), 9- and 13-hydroxy-octadedienoic acid (HODE)) decreases human EVCT invasion *in vitro* [12], [26], [38], [39]. Rosiglitazone belongs to the thiazolidinedione class of drugs, and is the first synthetic drug with high selectivity for the PPARγ isoform (*K*d approximately 40 nM). In transfection models, rosiglitazone does not activate the other PPARγ isoforms (PPARα and PPARδ) at concentrations as high as 100 µM [20]. As we have previously reported, inhibition of human primary EVCT invasion by rosiglitazone is concentration-dependent, with 50% inhibition at 1 µM [12]. This concentration has been widely used to activate PPARγ in various cellular models, including adipocytes and trophoblasts [40], [41]. In the 10-50 µM range, rosiglitazone has been reported to act through PPARγ-independent mechanisms [42], [43], [44].

Here, to identify differentially regulated genes that might play a role in placental trophoblast invasion, we used EVCTs isolated from 8–9 WA human placentas and compared gene expression patterns between these normally invasive EVCTs and rosiglitazone-treated EVCTs that display reduced invasiveness. Primary EVCTs were chosen because none of the available established human trophoblast cell lines display the same pattern of gene expression as primary cells [45]. The Achilles' heel of this model is the dearth of available cells.

The pleiotropic function of PPARγ in the placenta [46] is reflected by the diversity of regulated genes in rosiglitazone-treated EVCTs (Table S1). This regulation might be direct or indirect. Among the 20 genes containing a putative PPARγ response element (Figure 4), angiopoietin-like 4 (*ANGPTL4*), fatty acid binding protein 4 *(FABP4)* and plasminogen activator inhibitor 1 *(PAI-1, SERPINE1)* are direct PPARγ target genes [47], [48], [49].

Our findings highlight the differential regulation of several genes that might be involved in various aspects of placental development, including cell metabolism and invasive processes. The expression of numerous genes involved in fatty-acid metabolism and signaling was upregulated in rosiglitazone-treated EVCTs, including acyl-CoA synthetase long-chain family members 1 and 5 *(ACSL1, ACSL5)*, elongation of very long chain fatty acid like 4 *(ELOVL4)*, fatty acid-binding protein 4 and 5 (*FABP4, FABP5)*, insulin induced gene 1 *(INSIG1)* and very low density lipoprotein receptor *(VLDLR)* or downregulated as monoglyceride lipase *(MGLL)*. This supports PPARγ involvement in fatty acid metabolism in the human placenta, which is essential for normal fetal development [50], [51].

Cell invasion is a highly integrated multistep process responding to extracellular stimuli and involving cell adhesion and motility. It requires the coordination of a wide spectrum of signaling molecules and regulation of cytoskeleton dynamics [52]. Invasive cells move into neighboring tissue in a process that involves extracellular matrix degradation and proteolysis [53]. Among candidate genes that might be involved in the EVCT invasion process and that were differentially expressed in rosiglitazone-treated cells were several that encode proteinases, namely a disintegrin and metalloproteinase *(ADAM12)*, dipeptidylpeptidase 4 *(DPP4)* and pappalysin 2 *(PAPPA2)* or antiproteinase: PAI-1 *(PAI-1, SERPINE1)*. ADAM12 has extracellular metalloproteinase and cell-binding functions and intracellular signaling properties [54]. Extracellular proteolysis could have a proinvasive effect by affecting insulin-like growth factor (IGF) signaling through cleavage of IGF-binding proteins [55], [56] and epidermal growth factor (EGF) pathway *via* ectodomain shedding of membrane-tethered EGF-receptor ligands [57], [58]. Interaction of ADAM12 with cell-surface integrins may induce actin cytoskeletal changes. ADAM12 may also mediate signals through its intracellular domain (for review see [54]). DPP4 is a marker of EVCTs with a non invasive phenotype, and its down-regulation is associated with migration or invasion [59], [60]. Furthermore, DPP4 expression and activity are increased in preeclamptic placental tissues [61]. The metalloproteinase PAPPA2 cleaves IGFBP5 [62], potentially resulting in inhibition of EVCT invasion [63]. PAPPA2 is localized in the syncytiotrophoblast layer of placental villi and in invasive EVCTs [63], [64]. It is overexpressed at the maternal-fetal interface in placental samples from women with preeclampsia [63]. Concerning *PAI-1*, induction of its expression by TNF-α is associated with reduced EVCT invasion and migration in the first-trimester placental explant culture model [65].

A number of cytokines, growth factors and their receptors have been shown to regulate human trophoblast invasiveness [66], [67]. Here we observed differential expression of colony-stimulating factor 3 receptor *(CSF3R)*, insulin-like growth factor 1 receptor *(IGF1R)* and interleukin 10 receptor *(IL10RA)*. The granulocyte-colony-stimulating factor receptor (G-CSFR, CSF3R) is developmental stage-specific and has been detected in EVCTs *in situ* [68]. IGF-I has been shown to stimulate EVCT invasion through the α_v_β_3_ integrin signaling pathway [56]. IL10R is expressed by cytotrophoblasts at all gestational ages and its ligand (IL-10) has been reported to be an autocrine inhibitor of cytotrophoblast matrix metalloproteinase-9 (MMP-9) and invasion [69].

Many different molecules and signaling pathways coordinate cell migration, but dynamic cytoskeleton reorganization is at the heart of this process [36], [52]. This is illustrated here by the contraction of intermediate filaments in rosiglitazone-treated EVCTs (Figure 9). Rho family small guanosine triphosphate (GTP)-binding proteins (GTPases) coordinate the cellular responses required for cell migration by regulating the actin cytoskeleton and affecting the organization of the microtubule and intermediate filament networks, as well as cell-substrate adhesion [70]. The Rho signaling pathway has been implicated in trophoblast motility (for review see [36]). The expression of several genes encoding members of this signaling pathway was found to be modulated by rosiglitazone, including Rho GTPase-activating protein *(DLC1)*, Rho GTPase binding-protein *(CDC42EP4)* and Rho guanine nucleotide exchange factors (*ARHGEF3*, *ARHGEF4* and *NET1*). DLC1 is essential for embryonic development. In mice, *DLC1^−/−^* embryos do not survive beyond E10.5. Defects in the neural tube, brain, heart and placenta are observed, as well as altered organization of actin filaments and focal adhesions in cultured *DLC1^−/−^* fibroblasts [71]. Expression of *DLC-1* was strongly upregulated in rosiglitazone-treated EVCTs. *DLC1* is a potential tumor suppressor gene, as its expression reduces the migratory capacity of tumor cells [72]. In metastatic non-small-cell lung cancer cells (NSCLC), PPARγ overexpression has been shown to inhibit cell invasion and to be associated with DLC1 induction[73].

Gap junctional intercellular communication (GJIC) and connexin (Cx) expression are also involved in placental development [74], [75]. Connexins are a family of integral membrane proteins that oligomerize into clusters of intercellular channels called gap junctions. Gap junctions allow direct intercellular communication and diffusion of ions and signaling molecules between contacting cells. The expression of both Cx43 *(GJA1)* and Cx40 *(GJA5)* was upregulated in rosiglitazone-treated EVCTs. Cx43 is involved in human trophoblast differentiation and cell fusion [76], [77]. It has been detected in first-trimester villous trophoblasts and extravillous trophoblastic aggregated cells of the placental bed *in situ* [76], [78], and also in cultured EVCTs *in vitro* [79]. It is interesting to note that Cx43 expression in breast cancer cells reduces their metastasis to lung [80]. In the human placenta, Cx40 is expressed in proliferative EVCTs of the cell column. Expression becomes weak in distal cell columns when trophoblasts migrate; Cx40 is then re-expressed in trophoblasts aggregated within the decidua [76], [79]. According to Malassiné and Cronier, Cx40 plays a critical role in the switch from a proliferative to an invasive phenotype by trophoblastic cells invading the endometrium [75].

Heme oxygenase-1 (*HMOX-1*) was also upregulated in rosiglitazone-treated EVCTs. This corroborates the report from Bilban *et al.* [81] describing *HMOX-1* downregulation in human invasive EVCTs obtained from villous explant cultures by comparison with poorly invasive villous trophoblasts isolated from first-trimester placentas. Moreover, the latter authors identified *HMOX-1* as a negative regulator of trophoblast motility, acting *via* upregulation of PPARγ protein levels and activity in choriocarcinoma cell lines.

A number of trophoblastic genes were also downregulated following rosiglitazone treatment (Table S1) including *PPAR*γ itself as reported in murine trophoblast stem cells [41], suggesting the existence of a negative feedback loop.

Among the known PPARγ targets in the mouse trophoblast is the mucin gene *MUC1* [40]. In humans, *MUC1* is overexpressed in severe preeclampsia, and its overexpression suppresses trophoblast cell invasion [82]. Here, we found that *MUC1* expression was very weak (three probe sets, data not shown), in agreement with the work of Shyu *et al.* showing that *MUC1* mRNA and protein levels increase during human placental development and are barely detectable in first-trimester placentas [83].

Among the most strongly upregulated genes, we selected LOX for further investigation on the basis of published data, notably because its expression and activity are dependent on oxygen levels, and because it is known to have a role in tumor suppression, cell migration and invasion [30], [84], [85], [86], [87]. These properties indicate that LOX might play a role in trophoblast invasion during the first trimester of pregnancy when oxygen tension increases. Our analysis confirms earlier reports of the presence of LOX in human placenta *in situ* [88] and of LOXL1 transcripts in EVCTs *in vitro* [81]. We also detected LOXL2 transcripts in EVCT primary cultures, although protein expression was very low as reported by others at this stage of pregnancy [88]. *In vitro*, LOX and LOXL1 proteins were detected in EVCT primary cultures from 8- to 9-WA human chorionic villi. Confocal microscopy results further suggest that LOX protein could be concentrated in the cytoplasm, particularly in granules, while LOXL1 would be located mainly in the nuclei and nucleoli, probably after secretion, processing and internalization, as described by Hayashi K. *et al*. [89]. These observations are in keeping with the lower intracellular protein levels of LOX compared to LOXL1 in villi and EVCTs, as shown here by western blotting. In culture media, LOX and LOXL1 were detected as pro-proteins (about 50 kDa). The absence of the mature enzymes, produced by bone morphogenic protein (BMP) cleavage in the extracellular matrix [90], could be explained, in the case of LOXL1, by cellular uptake. The presence of LOX in the cell surroundings (immunocytochemistry) and in the insoluble fraction (western blot) suggests it may be trapped in the extracellular matrix secreted by EVCTs. The nuclear localization of LOXL1 suggests that it may have a role in the regulation of gene expression. Binding of LOX to histones H1 and H2 has been observed *in vitro* and *in vivo*, and has been suggested to modify the degree of chromatin compaction [91], [92]. The differential protein expression and subcellular localization of the LOX isoforms suggest that they could act through different mechanisms in EVCTs.

Inhibition of LOX enzymatic activity by BAPN enhanced the invasiveness of primary cultured EVCTs, while LOX upregulation by rosiglitazone was associated with a decrease in EVCT invasiveness. Furthermore, inhibition of EVCT invasiveness by rosiglitazone was totally overcome by BAPN treatment. Together, these findings suggest that the activity of LOX and/or LOXL1 negatively regulates cytotrophoblast invasion. LOX activity might reduce invasiveness through its ability to increase matrix stability.

LOX expression has been linked to both tumor progression and tumor suppression [93], [94]. In tumor cells, LOX activity promotes cell invasion and migration [30], [84], [86]. In contrast to active LOX, the LOX propeptide (LOX-PP) generated during the course of BMP-1-mediated LOX activation in the ECM acts as a tumor suppressor through multiple signaling pathways [85], [87].

Together, these observations suggest that LOXs regulate cell invasiveness through different mechanisms in normal and tumor cells. Thus, the available human invasive trophoblast cell lines, which consist of choriocarcinoma cells and transformed trophoblasts, are unlikely to mimic normal trophoblastic cell behavior, as pointed out by Bilban *et al*. [45].

The effects of global inhibition of LOX family activity by BAPN strongly suggest a role of LOXs in trophoblast invasion. This could be confirmed by selective inhibition of each LOX isoform with a siRNA strategy, such experiments remain to be performed.

In conclusion, our study provides the first transcriptome of PPARγ target genes in first-trimester primary cultures of human invasive EVCT, and supplies further evidence that trophoblast invasiveness is controlled by the PPARγ pathway, via novel downstream target genes. Among the latter, we show that LOX and/or LOXL1 activity may be involved in negative regulation of human trophoblast invasion.

## Materials and Methods

### Ethics statement

The study conformed to the Declaration of Helsinki. The placentas used for this study were obtained with the patients' written informed consent, and the protocol was approved by our local ethics committee (CCPRB Paris Cochin n° 18-05). Placental tissues were obtained from women undergoing legal and voluntary termination of a normal pregnancy during the 8-9th week of amenorrhea (WA), at Broussais hospital (Paris, France).

### EVCT isolation, purification and treatment

EVCTs were isolated from first-trimester chorionic villi (n = 6) as previously described [10], [11], with modifications. Cells were plated in culture dishes (Techno Plastic Products, Switzerland) coated with Matrigel™ (7 µg/cm^2^; Collaborative Biomedical Products, Le Pont de Claix, France) at 5×10^4^ cells/cm^2^ in DMEM-F12 culture medium containing Glutamax, 10% heat-inactivated fetal calf serum (FCS), 100 IU/mL penicillin and 100 µg/mL streptomycin (Invitrogen, Illkirch, France). The yield was up to 3.5×10^5^ non-proliferative EVCTs per gram of chorionic villi (up to 5 g at 8–9 WA). After 2 h at 37°C in a humidified incubator with 5% CO_2_, non-adherent cells were removed by washing. For cultures used in microarray experiments, RT-qPCR, immunocytochemistry and immunoblotting, the medium was replaced after a further 24 h and 1 µM rosiglitazone (Cayman, Ann Arbor, MI) dissolved at 1 mM in ethanol was added. There was no effect of 0.1% ethanol (vehicle) on EVCT cultures (data not shown). Rosiglitazone-treated and control cells were trypsinized 24 h later for RNA assays or 48 h later for protein assays.

### Microarray analysis

EVCT primary cultures from first-trimester placentas (rosiglitazone-treated and matched vehicle controls, n = 5 placentas) were obtained as described above. RNA was extracted using the TRIzol® reagent (Invitrogen) as described for RT-PCR, and then purified using the RNeasy® Mini kit (Quiagen S.A., Courtabœuf, France). RNA integrity and purity were checked using a 2100 Bioanalyzer with the RNA 6000 LabChip kit (Agilent Technologies, Massy, France). The GeneChip (U133A 2.0, Affymetrix, Inc., Santa Clara, CA), which analyzes 14 500 genes with 22 000 probe sets, was used according to the manufacturer's instructions. Data were processed with the Expression Analysis algorithm of the Affymetrix Microarray suite (version 4.0). CEL files were imported into BRB Array Tools software (http://linus.nci.nih.gov/BRB-ArrayTools.html) for normalization with the RMA algorithm and for further analysis. Differences in gene expression between roziglitazone-treated and vehicle control EVCTs were analyzed by using significance analysis of microarrays (SAM) [95] after applying a filtering threshold (i) less than 20% of expression data have at least 1.5-fold change in either direction from gene's median value; ii) percent of data missing or filtered out exceeds 50%) which selected 3354 probes. Statistical comparison was used to identify over- and under-expressed genes, focusing on genes that exhibited at least a 1.5-fold change, with a false discovery rate (FDR) of 5%.

### Functional analysis

Ingenuity Pathway Analysis software (IPA version 9.0, http://www.ingenuity.com/ , accessed 2011) was used to compute the statistical differences in gene sets between rosiglitazone treated and vehicule control EVCTs and to define cellular function and metabolic pathway involvement.

### Relative quantitative RT-PCR (RT-qPCR)

Relative RT-qPCR was used to compare the expression of selected genes in a new set of 6 independent experiments. Total RNA was extracted with the TRIzol® reagent, according to the manufacturer's instructions (Invitrogen). cDNA was synthesized from total RNA (0.5 µg) using the Superscript II Reverse Transcriptase kit (Invitrogen). All RT-qPCR reactions were performed in an ABI Prism 7300 Sequence Detection system (Applied Biosystems, Courtaboeuf, France) using a cDNA dilution corresponding to 6.25 ng of RNA with the SYBR® Green PCR kit (Applied Biosystems). Each experiment was performed in duplicate for each gene. An initial denaturation step at 95°C for 10 min was followed by 40 cycles at 95°C for 15 s and 60°C for 1 min, and by a three-step dissociation phase of 15 s at 95°C, 30 s at 60°C and 15 s at 95°C to eliminate possible artifacts such as oligonucleotide dimers. The primers used (Eurogentec, Angers, France) are described in Figure S2. Cytokeratin 7 (CK7), a trophoblast-specific intermediate filament in human placenta, and 18S RNA were used as endogenous RNA controls and gave similar results. Results normalized to 18S RNA are expressed as a percentage of the calibrated control (untreated sample).

### Absolute quantitative RT-qPCR

Absolute RT-qPCR was used to estimate the LOX isoform RNA copy number in 48-h primary EVCT cultures, using specific primers (Figure S2B). cDNAs of LOX and LOXL1 were obtained by reverse transcription of total RNA extracted from placental villi and amplification by Go Taq DNA polymerase (Promega, Madison, WI). The specificity of each DNA was determined by sequencing (Cogenics, Meylan, France). Amplified cDNA from three RT-PCR tubes for each isoform was purified using Nucleospin (Macherey-Nalgen, Düren, Germany) and the DNA concentration was measured with a Nanodrop® ND-1000 spectrophotometer. Copy numbers were determined by using the Avogadro number. Standard curves (10^2^ to 10^6^ cDNA copies) were prepared for each isoform in parallel with EVCT cDNA samples (n = 5), using the SYBR® Green PCR kit as described above (primers in Figure S2A).

### Immunoblotting

EVCTs were trypsinized after 72 h of culture, harvested, washed in PBS and frozen at -80°C. Proteins were extracted from EVCT pellets with the M-PER protein extraction reagent (Pierce) according to the manufacturer's instructions (http://www.piercenet.com/instructions/2160805.pdf). The M-PER pellet, representing the insoluble fraction (IF), was taken up in Laemmli buffer and sonicated [96]. Cell conditioned medium (CM) was collected from two dishes, centrifuged and then concentrated in a 10-kDa Ultra filter unit (Amicon Ultra, Millipore) by centrifuging at 3000 *g* for 20 min. Thirty and forty micrograms of protein from cell pellets and conditioned medium, respectively, and the whole insoluble fraction, were analyzed by western blot as previously described [97]. Twenty microliters of extracted Matrigel™ protein was run as a control. The Polyscreen polyvinylidene fluoride membrane (PerkinElmer, Courtaboeuf, France) was blocked in Tris-buffered saline, 0.1% Tween 20 (TBS-T) then incubated overnight at 4°C with specific rabbit polyclonal antibodies against LOX, LOXL1 or LOXL2 (1 µg/mL) [89], [96]. After three TBS-T washes, the membrane was incubated with a horseradish peroxidase-conjugated goat anti-rabbit IgG (0.03 µg/mL; Jackson ImmunoResearch Laboratories, West Grove, PA) for 1 h at room temperature. The specific bands were revealed by chemiluminescence (West Pico Chemiluminescent; Pierce) and visualized by autoradiography (Kodak Biomax MR film). After LOX, LOXL1 or LOXL2 immunoblotting, the membrane was reprobed with an anti-actin antibody (0.4 µg/mL; Sigma, Saint Quentin-Fallavier, France) as a control. Before reprobing, blots were stripped by incubation in 0.1 M 2-mercaptoethanol, 2% SDS, 62.5 mM Tris-HCl (pH 6.8) for 30 min at 50°C and then blocked in 5% nonfat milk, TBS-T for 30 min.

### Immunohistochemistry

Placenta samples (n = 3) were fixed in 4% paraformaldehyde (Electron Microscopy Sciences, Hatfield, PA) for 4 h at 4°C, then dehydrated and embedded in Paraplast®. Briefly, 5-µm placental tissue sections were deparaffinated in SafeSolv™ (Labonord, Templemars, France) and rehydrated in ethanol/water. Sections were permeabilized in PBS, 0.3% Triton X-100 for 4 min, and then washed 3 times in PBS for 5 min. Antigens were retrieved by treatment in citrate buffer, pH 6.1 (Dako, Trappes, France) for 40 min at 80°C. Non-specific binding was blocked by incubation for 15 min with PBS, 5% goat serum, 3% BSA, 22 µg/mL human IgG (Jackson ImmunoResearch Laboratories) at room temperature. Immunostaining was performed with a universal streptavidin–peroxidase immunostaining kit (LSAB®+Kit DAKO©, Trappes, France) using specific antibodies against LOX, LOXL1 or LOXL2 (2.5 µg/mL). Tissue sections were mounted in Dako Faramount aqueous mounting medium (Dako), then examined with an Olympus BX60 microscope (Olympus, Tokyo, Japan) and digitally photographed.

### Immunocytochemistry

EVCTs were isolated and cultured in 8-well Lab-Tek slides (Nunc, Dutscher, Brumath, France) (4×10^4^ cells/well) as described above. The cultures were washed with PBS containing Ca^2+^ and Mg^2+^, fixed with 4% paraformaldehyde [98] and permeabilized for 10 min in PBS, 0.1% Triton X-100. After washing in PBS, saturation was achieved with a blocking solution (PBS, 5% donkey serum, 12.5 µg/mL human IgG) for 1 h at room temperature. LOXs proteins were immunodetected with the same antibodies as above, overnight at 4°C, followed by a mouse monoclonal anti-CK7 (1.4 µg/mL; clone OV-TL 12/30, Dako) for 1 h at room temperature. A non-specific IgG was used as a control at the same concentration as the primary antibody. Secondary antibodies were fluorescein isothiocyanate-conjugated (FITC) donkey anti-rabbit (3 µg/mL) and CY3-conjugated anti-mouse (5 µg/mL; Jackson ImmunoResearch Laboratories) [99]. Nuclei were labeled with 1 µM Topro®-3 iodide (Molecular Probes, Invitrogen) for 10 min at room temperature, followed by two PBS washes. The slides were mounted with Vectashield® (Vector Laboratories, Burlingame, CA) and examined with a Leica confocal microscope (IFR71-IMTCE Imaging Facility, Paris Descartes University).

### Invasion assay

EVCTs (25×10^4^ cells), isolated as described above, were plated in the upper chamber of 8-micron Transwell inserts (6.5 mm diameter; BD Falcon, le Pont de Claix, France) coated with 60 µg Matrigel™ and incubated for 2 hours in EVCT culture medium. The cells were then washed and treated for 48 h with complete medium containing 100 or 200 µM BAPN (Sigma), a specific LOX inhibitor [33]; BAPN stock solution was 100 mM in water. Medium without BAPN was used as a control. The cells were washed and fixed with 4% paraformaldehyde for 20 min at room temperature then visualized by immunostaining with anti-CK7 as described above. The filters were cut out from the inserts and placed on a glass slide. Nuclei were labeled with fluorescent 4′,6-diamino-2-phenylindole (DAPI) mounting medium (Vector Laboratories) and the cells were examined with an Olympus BX60 epifluorescence microscope. Images were captured with a Hamamatsu C4742-95 CCD camera and VisionStage® software (Alliance Vision, Montelimar, France). Ten representative fields were photographed for each filter. Cells and pseudopods were counted manually and the number of migrating cells was divided by the total number of nuclei (DAPI stain). The numbers of invasive cells in treated cultures are expressed as a percentage of control cultures, and are the mean of four separate experiments performed in duplicate.

### Statistical analysis

Values reported here are the mean and SEM of 4–6 primary cultures, each derived from a different placenta. ANOVA and the Mann-Whitney test were used to compare treated and control EVCT cultures for mRNA expression, the number of *LOX* isoform RNA copies and invasive capacity. Differences were considered significant when p<0.05 (ANOVA).

## Supporting Information

Figure S1 **Heatmap of the 175 probe sets (139 genes, 117 unique genes) selected with the SAM procedure, together with the probes and gene names.**
(TIF)

Figure S2 **Forward and reverse oligonucleotides used for absolute and relative qPCR.**
(TIF)

Table S1 **The complete list of upregulated and downregulated genes in rosiglitazone-treated EVCTs.**
(DOC)
