# Supplementary figures and images for: Transcriptome Analysis of PPARγ Target Genes Reveals the Involvement of Lysyl Oxidase in Human Placental Cytotrophoblast Invasion
Source: PLoS One. 2013 Nov 12;8(11):e79413. doi: 10.1371/journal.pone.0079413 (PMC3827157; doi:10.1371/journal.pone.0079413)

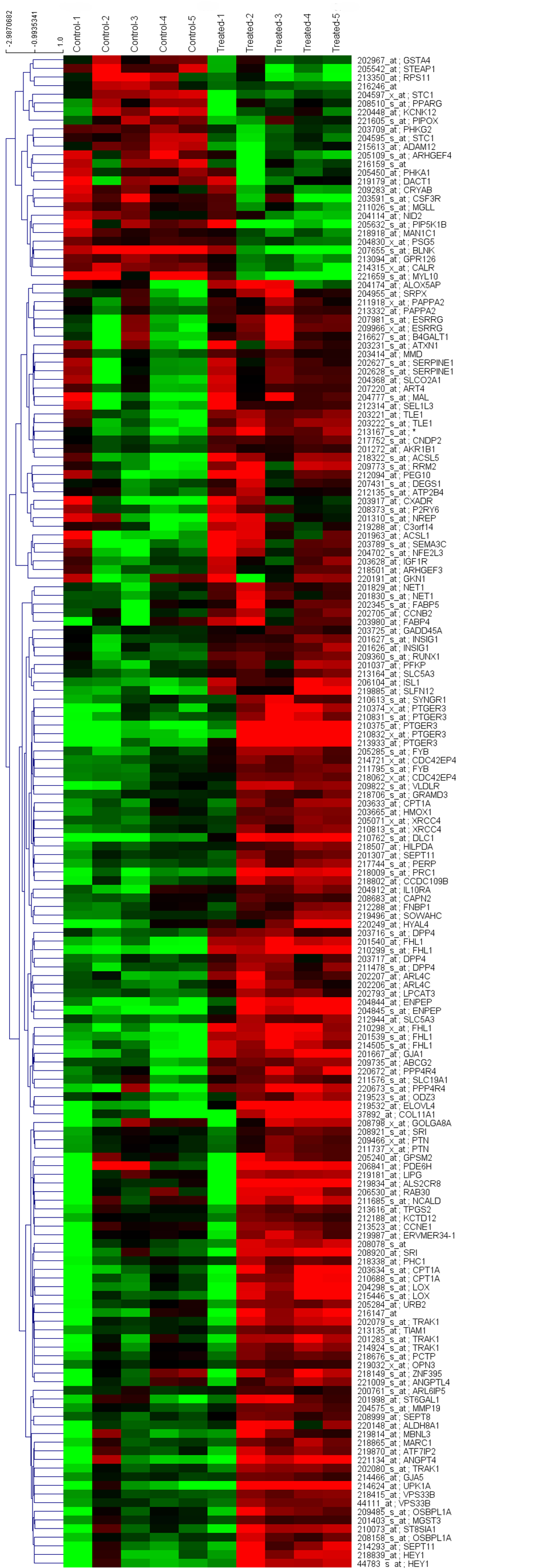

Supplement: Figure S1 — Heatmap of the 175 probe sets (139 genes, 117 unique genes) selected with the SAM procedure, together with the probes and gene names. (TIF) [file pone.0079413.s001.tif]

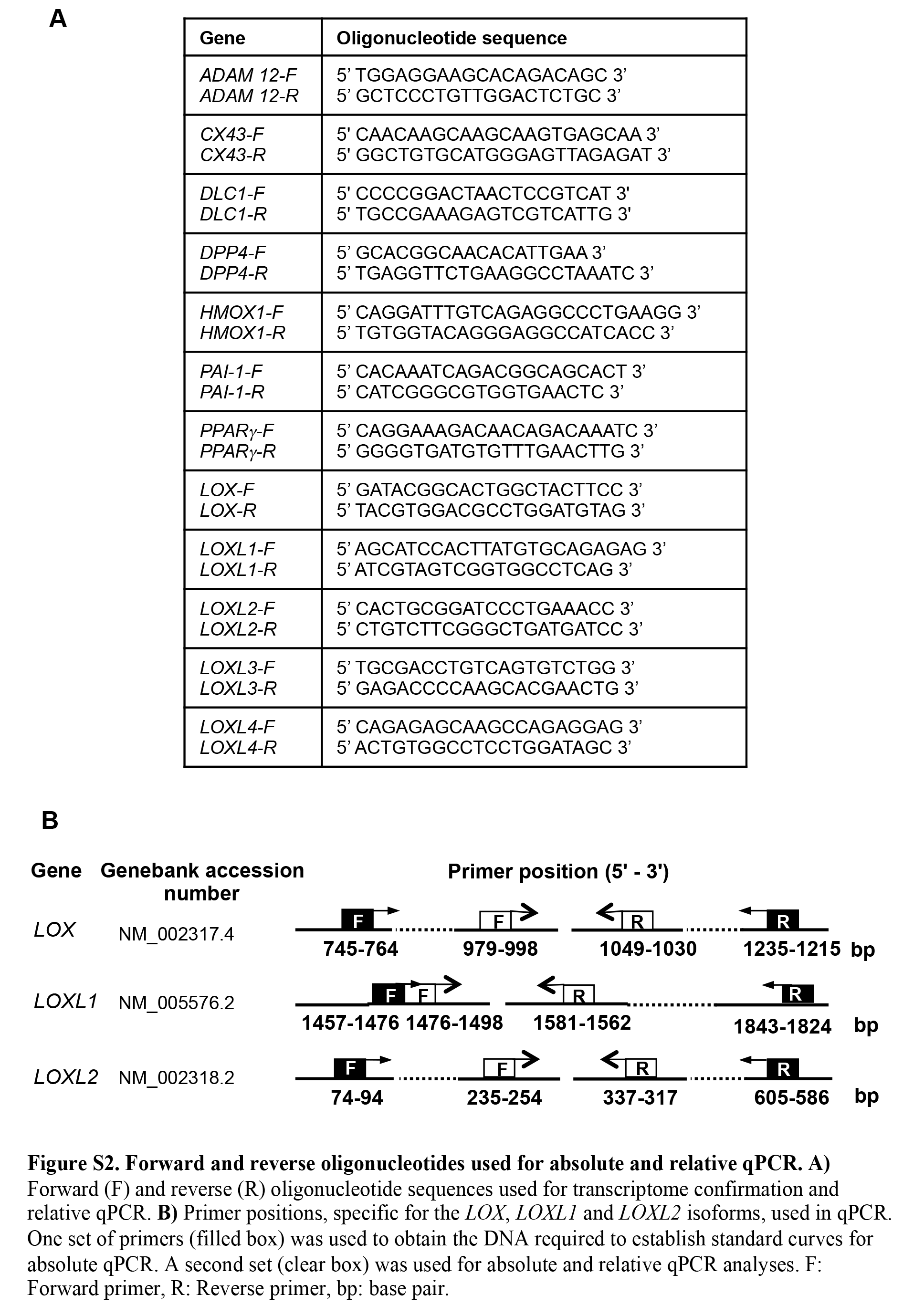

Supplement: Figure S2 — Forward and reverse oligonucleotides used for absolute and relative qPCR. (TIF) [file pone.0079413.s002.tif]
