# Supplementary material for: Transcriptome Analysis of PPARγ Target Genes Reveals the Involvement of Lysyl Oxidase in Human Placental Cytotrophoblast Invasion
Source: PLoS One. 2013 Nov 12;8(11):e79413. doi: 10.1371/journal.pone.0079413 (PMC3827157; doi:10.1371/journal.pone.0079413)
Supplement: Table S1 — The complete list of upregulated and downregulated genes in rosiglitazone-treated EVCTs. (DOC) [file pone.0079413.s003.doc]

**Supplemental table 1**

**Complete list of up-regulated and down-regulated genes in rosiglitazone-treated EVCTs**

| **Fold change (treated/control)** | **Probe set** | **Description** | **gene symbol** |
| --- | --- | --- | --- |
| 10 | 210762_s_at | deleted in liver cancer 1 | DLC1 |
| 10 | 219532_at | elongation of very long chain fatty acids (FEN1/Elo2. SUR4/Elo3. yeast)-like 4 | ELOVL4 |
| 5.00 | 213933_at | prostaglandin E receptor 3 (subtype EP3) | PTGER3 |
| 5.00 | 210375_at | prostaglandin E receptor 3 (subtype EP3) | PTGER3 |
| 5.00 | 201667_at | gap junction protein. alpha 1. 43kDa (connexin 43) | GJA1 |
| 3.33 | 201540_at | four and a half LIM domains 1 | FHL1 |
| 3.33 | 210298_x_at | four and a half LIM domains 1 | FHL1 |
| 3.33 | 210299_s_at | four and a half LIM domains 1 | FHL1 |
| 3.33 | 201539_s_at | four and a half LIM domains 1 | FHL1 |
| 3.33 | 214505_s_at | four and a half LIM domains 1 | FHL1 |
| 3.33 | 210832_x_at | prostaglandin E receptor 3 (subtype EP3) | PTGER3 |
| 3.33 | 209735_at | ATP-binding cassette. sub-family G (WHITE). member 2 | ABCG2 |
| 3.33 | 221134_at | angiopoietin 4 | ANGPT4 |
| 3.33 | 204298_s_at | lysyl oxidase | LOX |
| 3.33 | 214624_at | uroplakin 1A | UPK1A |
| 3.33 | 215446_s_at | lysyl oxidase | LOX |
| 3.33 | 203980_at | fatty acid binding protein 4. adipocyte | FABP4 |
| 2.50 | 204777_s_at | mal. T-cell differentiation protein | MAL |
| 2.50 | 220249_at | hyaluronoglucosaminidase 4 | HYAL4 |
| 2.50 | 208078_s_at |  |  |
| 2.50 | 210374_x_at | prostaglandin E receptor 3 (subtype EP3) | PTGER3 |
| 2.50 | 44783_s_at | hairy/enhancer-of-split related with YRPW motif 1 | HEY1 |
| 2.50 | 218802_at | coiled-coil domain containing 109B | CCDC109B |
| 2.50 | 218839_at | hairy/enhancer-of-split related with YRPW motif 1 | HEY1 |
| 2.50 | 220673_s_at | protein phosphatase 4, regulatory subunit 4 | PPP4R4 |
| 2.50 | 208920_at | sorcin | SRI |
| 2.50 | 218322_s_at | acyl-CoA synthetase long-chain family member 5 | ACSL5 |
| 2.50 | 201037_at | phosphofructokinase. platelet | PFKP |
| 2.50 | 212944_at | solute carrier family 5 (inositol transporters), member 3 | SLC5A3 |
| 2.50 | 209822_s_at | very low density lipoprotein receptor | VLDLR |
| 2.50 | 218009_s_at | protein regulator of cytokinesis 1 | PRC1 |
| 2.50 | 207981_s_at | estrogen-related receptor gamma | ESRRG |
| 2.50 | 202628_s_at | serpin peptidase inhibitor. clade E (nexin. plasminogen activator inhibitor type 1). member 1 | SERPINE1 |
| 2.50 | 204368_at | solute carrier organic anion transporter family. member 2A1 | SLCO2A1 |
| 2.50 | 202627_s_at | serpin peptidase inhibitor. clade E (nexin. plasminogen activator inhibitor type 1). member 1 | SERPINE1 |
| 2.50 | 204844_at | glutamyl aminopeptidase (aminopeptidase A) | ENPEP |
| 2.50 | 203221_at | transducin-like enhancer of split 1 (E(sp1) homolog. Drosophila) | TLE1 |
| 2.50 | 201963_at | acyl-CoA synthetase long-chain family member 1 | ACSL1 |
| 2.50 | 202345_s_at | fatty acid binding protein 5 (psoriasis-associated) | FABP5 |
| 2.50 | 37892_at | collagen. type XI. alpha 1 | COL11A1 |
| 2.50 | 219523_s_at | odz. odd Oz/ten-m homolog 3 (Drosophila) | ODZ3 |
| 2.00 | 202207_at | ADP-ribosylation factor-like 4C | ARL4C |
| 2.00 | 218507_at | hypoxia inducible lipid droplet-associated | HILPDA |
| 2.00 | 211795_s_at | FYN binding protein (FYB-120/130) | FYB |
| 2.00 | 204575_s_at | matrix metallopeptidase 19 | MMP19 |
| 2.00 | 210073_at | ST8 alpha-N-acetyl-neuraminide alpha-2.8-sialyltransferase 1 | ST8SIA1 |
| 2.00 | 202079_s_at | trafficking protein, kinesin binding 1 | TRAK1 |
| 2.00 | 212094_at | paternally expressed 10 | PEG10 |
| 2.00 | 214466_at | gap junction protein. alpha 5. 40kDa (connexin 40) | GJA5 |
| 2.00 | 210831_s_at | prostaglandin E receptor 3 (subtype EP3) | PTGER3 |
| 2.00 | 203716_s_at | dipeptidylpeptidase 4 (CD26. adenosine deaminase complexing protein 2) | DPP4 |
| 2.00 | 203665_at | heme oxygenase (decycling) 1 | HMOX1 |
| 2.00 | 217744_s_at | PERP. TP53 apoptosis effector | PERP |
| 2.00 | 213164_at | solute carrier family 5 (inositol transporters). member 3 | SLC5A3 |
| 2.00 | 204845_s_at | glutamyl aminopeptidase (aminopeptidase A) | ENPEP |
| 2.00 | 206104_at | ISL1 transcription factor. LIM/homeodomain. (islet-1) | ISL1 |
| 2.00 | 201998_at | ST6 beta-galactosamide alpha-2.6-sialyltranferase 1 | ST6GAL1 |
| 2.00 | 214293_at | septin 11 | SEPT11 |
| 2.00 | 213167_s_at |  |  |
| 2.00 | 219496_at | sosondowah ankyrin repeat domain family member C | SOWAHC |
| 2.00 | 201283_s_at | trafficking protein, kinesin binding 1 | TRAK1 |
| 2.00 | 202080_s_at | trafficking protein, kinesin binding 1 | TRAK1 |
| 2.00 | 205285_s_at | FYN binding protein (FYB-120/130) | FYB |
| 2.00 | 204174_at | arachidonate 5-lipoxygenase-activating protein | ALOX5AP |
| 2.00 | 209360_s_at | runt-related transcription factor 1 (acute myeloid leukemia 1; aml1 oncogene) | RUNX1 |
| 2.00 | 217752_s_at | CNDP dipeptidase 2 (metallopeptidase M20 family) | CNDP2 |
| 2.00 | 203633_at | carnitine palmitoyltransferase 1A (liver) | CPT1A |
| 2.00 | 211478_s_at | dipeptidylpeptidase 4 (CD26. adenosine deaminase complexing protein 2) | DPP4 |
| 2.00 | 219834_at | amyotrophic lateral sclerosis 2 (juvenile) chromosome region. candidate 8 | ALS2CR8 |
| 2.00 | 201626_at | insulin induced gene 1 | INSIG1 |
| 2.00 | 216147_at |  |  |
| 2.00 | 204912_at | interleukin 10 receptor. alpha | IL10RA |
| 2.00 | 203222_s_at | transducin-like enhancer of split 1 (E(sp1) homolog. Drosophila) | TLE1 |
| 2.00 | 201829_at | neuroepithelial cell transforming gene 1 | NET1 |
| 2.00 | 209773_s_at | ribonucleotide reductase M2 polypeptide | RRM2 |
| 2.00 | 219885_at | schlafen family member 12 | SLFN12 |
| 2.00 | 203634_s_at | carnitine palmitoyltransferase 1A (liver) | CPT1A |
| 2.00 | 206841_at | phosphodiesterase 6H. cGMP-specific. cone. gamma | PDE6H |
| 2.00 | 201830_s_at | neuroepithelial cell transforming gene 1 | NET1 |
| 1.67 | 208158_s_at | oxysterol binding protein-like 1A | OSBPL1A |
| 1.67 | 214721_x_at | CDC42 effector protein (Rho GTPase binding) 4 | CDC42EP4 |
| 1.67 | 202793_at | lysophosphatidylcholine acyltransferase 3 | LPCAT3 |
| 1.67 | 201403_s_at | microsomal glutathione S-transferase 3 | MGST3 |
| 1.67 | 218062_x_at | CDC42 effector protein (Rho GTPase binding) 4 | CDC42EP4 |
| 1.67 | 211576_s_at | solute carrier family 19 (folate transporter). member 1 | SLC19A1 |
| 1.67 | 213332_at | Pappalysin 2 | PAPPA2 |
| 1.67 | 211685_s_at | neurocalcin delta | NCALD |
| 1.67 | 214924_s_at | trafficking protein, kinesin binding 1 | TRAK1 |
| 1.67 | 201272_at | aldo-keto reductase family 1. member B1 (aldose reductase) | AKR1B1 |
| 1.67 | 201307_at | septin 11 | SEPT11 |
| 1.67 | 218501_at | rho guanine nucleotide exchange factor (GEF) 3 | ARHGEF3 |
| 1.67 | 213135_at | T-cell lymphoma invasion and metastasis 1 | TIAM1 |
| 1.67 | 44111_at | vacuolar protein sorting 33B (yeast) | VPS33B |
| 1.67 | 218706_s_at | GRAM domain containing 3 | GRAMD3 |
| 1.67 | 213523_at | cyclin E1 | CCNE1 |
| 1.67 | 218415_at | vacuolar protein sorting 33B (yeast) | VPS33B |
| 1.67 | 219814_at | muscleblind-like 3 (Drosophila) | MBNL3 |
| 1.67 | 206530_at | RAB30. member RAS oncogene family | RAB30 |
| 1.67 | 205071_x_at | X-ray repair complementing defective repair in Chinese hamster cells 4 | XRCC4 |
| 1.67 | 210813_s_at | X-ray repair complementing defective repair in Chinese hamster cells 4 | XRCC4 |
| 1.67 | 219987_at | endogenous retrovirus group MER34, member 1 | ERVMER34-1 |
| 1.67 | 204702_s_at | nuclear factor (erythroid-derived 2)-like 3 | NFE2L3 |
| 1.67 | 208999_at | septin 8 | SEPT08 |
| 1.67 | 209966_x_at | estrogen-related receptor gamma | ESRRG |
| 1.67 | 212314_at | sel-1 suppressor of lin-12-like 3 (C. elegans) | SEL1L3 |
| 1.67 | 210688_s_at | carnitine palmitoyltransferase 1A (liver) | CPT1A |
| 1.67 | 211918_x_at | pappalysin 2 | PAPPA2 |
| 1.67 | 218676_s_at | phosphatidylcholine transfer protein | PCTP |
| 1.67 | 203789_s_at | sema domain. immunoglobulin domain (Ig). short basic domain. secreted. (semaphorin) 3C | SEMA3C |
| 1.67 | 218865_at | mitochondrial amidoxime reducing component 1 | MARC1 |
| 1.67 | 203717_at | dipeptidylpeptidase 4 (CD26. adenosine deaminase complexing protein 2) | DPP4 |
| 1.67 | 211737_x_at | pleiotrophin | PTN |
| 1.67 | 203231_s_at | ataxin 1 | ATXN1 |
| 1.67 | 207431_s_at | degenerative spermatocyte homolog 1. lipid desaturase (Drosophila) | DEGS1 |
| 1.67 | 207220_at | ADP-ribosyltransferase 4 (Dombrock blood group) | ART4 |
| 1.67 | 203628_at | insulin-like growth factor 1 receptor | IGF1R |
| 1.67 | 218149_s_at | zinc finger protein 395 | ZNF395 |
| 1.67 | 204955_at | sushi-repeat-containing protein. X-linked | SRPX |
| 1.67 | 221009_s_at | angiopoietin-like 4 | ANGPTL4 |
| 1.67 | 219181_at | lipase. endothelial | LIPG |
| 1.67 | 219870_at | activating transcription factor 7 interacting protein 2 | ATF7IP2 |
| 1.67 | 201627_s_at | insulin induced gene 1 | INSIG1 |
| 1.67 | 209485_s_at | oxysterol binding protein-like 1A | OSBPL1A |
| 1.67 | 220672_at | protein phosphatase 4, regulatory subunit 4 | PPP4R4 |
| 1.67 | 201310_s_at | neuronal regeneration related protein | NREP |
| 1.67 | 208921_s_at | sorcin | SRI |
| 1.67 | 203414_at | monocyte to macrophage differentiation-associated | MMD |
| 1.67 | 218338_at | polyhomeotic-like 1 (Drosophila) | PHC1 |
| 1.67 | 208683_at | calpain 2. (m/II) large subunit | CAPN2 |
| 1.67 | 202206_at | ADP-ribosylation factor-like 7 | ARL4C |
| 1.67 | 210613_s_at | synaptogyrin 1 | SYNGR1 |
| 1.67 | 220148_at | aldehyde dehydrogenase 8 family. member A1 | ALDH8A1 |
| 1.67 | 212288_at | formin binding protein 1 | FNBP1 |
| 1.43 | 202705_at | cyclin B2 | CCNB2 |
| 1.43 | 219288_at | chromosome 3 open reading frame 14 | C3orf14 |
| 1.43 | 203917_at | coxsackie virus and adenovirus receptor | CXADR |
| 1.43 | 212188_at | potassium channel tetramerisation domain containing 12 | KCTD12 |
| 1.43 | 220191_at | gastrokine 1 | GKN1 |
| 1.43 | 208373_s_at | pyrimidinergic receptor P2Y. G-protein coupled. 6 | P2RY6 |
| 1.43 | 219032_x_at | opsin 3 (encephalopsin. panopsin) | OPN3 |
| 1.43 | 205240_at | G-protein signalling modulator 2 (AGS3-like. C. elegans) | GPSM2 |
| 1.43 | 212135_s_at | ATPase. Ca++ transporting. plasma membrane 4 | ATP2B4 |
| 1.43 | 200761_s_at | ADP-ribosylation-like factor 6 interacting protein 5 | ARL6IP5 |
| 1.43 | 209466_x_at | pleiotrophin | PTN |
| 1.43 | 213616_at | tubulin polyglutamylase complex subunit 2 | TPGS2 |
| 1.43 | 205284_at | URB2 ribosome biogenesis 2 homolog (S. cerevisiae) | URB2 |
| 1.43 | 208798_x_at | golgi autoantigen. golgin subfamily a. 8A | GOLGA8A |
| 1.43 | 203725_at | growth arrest and DNA-damage-inducible. alpha | GADD45A |
| 1.43 | 216627_s_at | UDP-Gal:betaGlcNAc beta 1.4- galactosyltransferase. polypeptide 1 | B4GALT1 |
| 0.67 | 205450_at | phosphorylase kinase. alpha 1 (muscle) | PHKA1 |
| 0.63 | 218918_at | mannosidase. alpha. class 1C. member 1 | MAN1C1 |
| 0.59 | 221605_s_at | pipecolic acid oxidase | PIPOX |
| 0.56 | 216159_s_at |  |  |
| 0.56 | 219179_at | dapper. antagonist of beta-catenin. homolog 1 (Xenopus laevis) | DACT1 |
| 0.56 | 209283_at | crystallin. alpha B | CRYAB |
| 0.56 | 204595_s_at | stanniocalcin 1 | STC1 |
| 0.56 | 205109_s_at | rho guanine nucleotide exchange factor (GEF) 4 | ARHGEF4 |
| 0.56 | 211026_s_at | monoglyceride lipase | MGLL |
| 0.53 | 204597_x_at | stanniocalcin 1 | STC1 |
| 0.53 | 204114_at | nidogen 2 (osteonidogen) | NID2 |
| 0.53 | 203591_s_at | colony stimulating factor 3 receptor (granulocyte) | CSF3R |
| 0.53 | 203709_at | phosphorylase kinase. gamma 2 (testis) | PHKG2 |
| 0.53 | 208510_s_at | peroxisome proliferative activated receptor. gamma | PPARG |
| 0.53 | 205542_at | six transmembrane epithelial antigen of the prostate 1 | STEAP1 |
| 0.53 | 204830_x_at | pregnancy specific beta-1-glycoprotein 5 | PSG5 |
| 0.50 | 202967_at | glutathione S-transferase A4 | GSTA4 |
| 0.50 | 205632_s_at | phosphatidylinositol-4-phosphate 5-kinase. type I. beta | PIP5K1B |
| 0.48 | 216246_at |  |  |
| 0.45 | 215613_at | ADAM metallopeptidase domain 12 (meltrin alpha) | ADAM12 |
| 0.42 | 220448_at | potassium channel. subfamily K. member 12 | KCNK12 |
| 0.42 | 213094_at | G protein-coupled receptor 126 | GPR126 |
| 0.36 | 214315_x_at | calreticulin | CALR |
| 0.33 | 207655_s_at | B-cell linker | BLNK |
| 0.24 | 213350_at | ribosomal protein S11 | RPS11 |
| 0.19 | 221659_s_at | myosin, light chain 10, regulatory | MYL10 |
